# From Major Chapple, M.P., R.A.M.C.

**Published:** 1918-08-03

**Authors:** W. A. Chapple

**Affiliations:** House of Commons.


### THE LONDON HOSPITAL NURSES.

**[The two following letters have appeared in "The Times" since our last issue, in addition to previous correspondence, see "The Hospital," p. 375.]**

### From Major Chapple, M.P., R.A.M.C.

Sir,—Lord Knutsford has replied to my challenge, "I do deny the truth of his statements." Here is the proof that they are true.

1. "Two years of service are required after the two years of training . . ." (Section XLVI., London Hospital Standing Orders).

"Nurses are not sent out now until they have done two years in the hospital except in exceptional circumstances." (Lord Knutsford, Select Committee on Registration of Nurses, 1904.)

2. (*a*) "Dr. Chapple's other charge . . . that we exploit the nurses by paying them 11s. 6d. a week when we send them out and charging the patients £2 2s. He does not state . . . all the other advantages they enjoy." (E. W. Morris, secretary, London Hospital, in the *Standard*, March, 1914.) Recently the nurse's pay has been raised to 13s. 5d. per week and the charge to private patients to a minimum of £2 12s. 6d.—a war rise of 1s. 11d. per week to the nurse, while she is earning an extra 8s. 7d. per week for her employer. (*b*) "We pay our private staff from £35 (13s. 5d. per week) to £55 per year, and 2s. 6d. a week for washing." (Lord Knutsford in the *Times*.) (*c*) "I had to pay £3 13s. 6d. a week . . . I also paid 2s. 6d. for her washing." (A correspondent.) (*d*) "She was sent from the ward to my residence, and on arrival presented me with papers showing the hospital charge for her services was £2 2s. per week, plus 2s. 6d. for laundry, and all fares, etc., extra. . . . After eight weeks in all the fees were still further advanced to £3 13s. 6d. . . . and this rate continued until the end of 12 weeks." (J. P. Lucas, a correspondent.)

3. "All these things have got to be put against the profit of £5,000 a year which we make for sending out nurses on private work." (E. W. Morris, secretary, London Hospital, in the *Standard*, March, 1914.)

I saw the official figures in a London Hospital report, which showed a profit in one year before the war of over £6,000.—Yours faithfully,

House of Commons. W. A. Chapple.

